# A nucleotide-controlled conformational switch modulates the activity of eukaryotic IMP dehydrogenases

**DOI:** 10.1038/s41598-017-02805-x

**Published:** 2017-06-01

**Authors:** Rubén M. Buey, David Fernández-Justel, Íñigo Marcos-Alcalde, Graeme Winter, Paulino Gómez-Puertas, José María de Pereda, José Luis Revuelta

**Affiliations:** 1 Metabolic Engineering Group, Dpto. Microbiología y Genética, Universidad de Salamanca, Campus Miguel de Unamuno, 37007 Salamanca, Spain; 2 Molecular Modelling Group, Centro de Biología Molecular “Severo Ochoa” (CSIC-UAM), ES-28049 Madrid, Spain; 3 Diamond Light Source, Harwell Science and Innovation Campus, Didcot, Oxfordshire OX11 0DE England UK; 4 Instituto de Biología Molecular y Celular del Cáncer (ICSIC-Universidad de Salamanca), Campus Miguel de Unamuno, 37007 Salamanca, Spain

## Abstract

Inosine-5′-monophosphate dehydrogenase (IMPDH) is an essential enzyme for nucleotide metabolism and cell proliferation. Despite IMPDH is the target of drugs with antiviral, immunosuppressive and antitumor activities, its physiological mechanisms of regulation remain largely unknown. Using the enzyme from the industrial fungus *Ashbya gossypii*, we demonstrate that the binding of adenine and guanine nucleotides to the canonical nucleotide binding sites of the regulatory Bateman domain induces different enzyme conformations with significantly distinct catalytic activities. Thereby, the comparison of their high-resolution structures defines the mechanistic and structural details of a nucleotide-controlled conformational switch that allosterically modulates the catalytic activity of eukaryotic IMPDHs. Remarkably, retinopathy-associated mutations lie within the mechanical hinges of the conformational change, highlighting its physiological relevance. Our results expand the mechanistic repertoire of Bateman domains and pave the road to new approaches targeting IMPDHs.

## Introduction

Inosine-5′-monophosphate dehydrogenase (IMPDH; EC 1.1.1.205) catalyzes the oxidative reaction of IMP to xanthosine 5′-monophosphate (XMP), the rate-limiting step in the *de novo* synthesis of guanine nucleotides. Thereby, IMPDH plays an essential role in the regulation of the intracellular purine nucleotide pools. IMPDH inhibition results in a strong reduction of the intracellular guanine nucleotide levels and the subsequent imbalance between the guanine and adenine nucleotide pools with dramatic consequences for cell proliferation^1^. Consequently, IMPDH is the cellular target of a diverse family of drugs widely used in clinical chemotherapy as antivirals, immunosuppressive or antitumor agents^2^. Due to its clinical relevance, IMPDH has been extensively studied during the last two decades and a significant amount of information is available with respect to the catalytic mechanism and its inhibition mediated by drugs^3^. However, there is an evident lack of knowledge on the physiological regulation of IMPDH that only recently has started to focus attention^4, 5^.

Most IMPDHs are homotetramers in solution with each monomer composed of a catalytic and a regulatory domain (Supplementary Fig. 1). The catalytic domain is an archetypal TIM barrel^6^ with an especial feature consisting of a twisted beta sheet, called “finger domain”^7^, that projects outwards from the carboxy terminal face of the β-barrel. The finger domain is present in all known IMPDHs and is essential for the catalytic activity^5^. Within the finger domain there exists a loop, called “catalytic flap” that moves into the active site during the catalytic cycle (when NADH departs) to properly position the catalytic residues in order to complete the enzymatic reaction^3, 8^. Additionally, the finger domains of IMPDH have been implied in allosteric regulation because, despite its conformation does not significantly change upon GTP/GDP inhibition, the alteration of their internal dynamics and flexibility results in a decrease of the affinity for the substrate IMP^5^. The regulatory module consists of two repeats of the cystathionine β-synthase domain (constituting a CBS pair or Bateman domain^9^) that are inserted within a loop of the catalytic domain. Bateman domains form a large and widely distributed domain superfamily that act as allosteric modulators of diverse protein functions in response to the binding of different ligands. Indeed, Bateman domains are modules that sense the cellular energy status, metal ion concentration or ionic strength and regulate enzymatic activity accordingly^10, 11^. The relevance of Bateman domains is stressed by the fact that mutations in them are associated to a variety of human hereditary diseases, including the Wolff-Parkinson-White syndrome, Congenital Myotonia, Homocystinuria, etc.^12, 13^. In IMPDH, missense mutations within the Bateman domain of human isoform 1 (HsIMPDH1) are linked to Leber Congenital Amaurosis and Retinitis Pigmentosa^14^.

The Bateman domain is dispensable for the catalytic activity of IMPDHs^4, 15–19^, but is essential in a number of functions associated to IMDPH. It has been associated to the recognition of single stranded DNA^20^ and the regulation of transcription^21^, to the modulation of cell growth through the interaction with the polyketide sanglifehrin A^22^, as well as to the allosteric regulation of the catalytic activity by purine nucleotides of eukaryotic^5, 23^ and prokaryotic^4, 24^ IMPDHs.

The Bateman domains of eukaryotic IMPDHs bind three guanine nucleotides: two of them in the canonical nucleotide binding sites and a third one in a non-canonical site^5^. GTP and GDP binding to the Bateman domain of eukaryotic IMPDHs induces a tail-to-tail dimerization of tetramers, forcing the finger domains of both tetramers to interact, thus resulting in octamers with compromised catalytic activity. In contrast, adenine nucleotides do not significantly affect the catalytic activity of eukaryotic IMPDHs^5, 20, 23, 25^. On the other hand, the Bateman domains of bacterial IMPDHs bind two ATP molecules in the canonical nucleotide binding sites^4^, but they do not bind guanine nucleotides^5, 26^. According to a recent classification, class-I bacterial IMPDHs are auto-inhibited i*n vitro* in the absence of nucleotides and require ATP to achieve full catalytic activity^24^. In contrast, class-II bacterial IMDPHs are active in the absence of nucleotides and, thereby, do not need ATP to achieve full activity^5, 24^. These data demonstrate that eukaryotic and prokaryotic IMPDHs have evolved different allosteric regulatory mechanisms that allow adapting to the metabolic requirements of each particular organism.

Nonetheless, despite these recent reports on the allosteric regulation of IMPDHs by purine nucleotides, the mechanistic and structural details of the communication between the Bateman and catalytic domains remain mostly unclear. Indeed, a major goal in the field is the determination of high-resolution full-length structure pairs of active and inactive IMPDH enzymes that allow unveiling the intramolecular signaling pathways and the structural basis of allosteric regulation.

In this study, using the IMPDH enzyme from the industrial fungus *Ashbya gossypii* (AgIMPDH) as a model, we show that both adenine (ATP, ADP and AMP) and guanine (GDP and GTP) nucleotides bind to and alter the structure of IMPDH. Adenine and guanine nucleotides compete for the canonical binding sites of the Bateman domain, inducing different IMPDH conformations with significantly different catalytic activity. Thereby, the comparison of the high-resolution structures of the activated (in complex with ATP) and inhibited (in complex with either GDP^5^ or a mixture of ATP/GDP) AgIMPDH defines the atomic and mechanistic details of a novel purine-nucleotide-controlled conformational switch that allosterically modulate the catalytic activity of eukaryotic IMPDHs. Remarkably, several residues in AgIMPDH that correspond to missense mutations in HsIMPDH1 associated to human retinopathies^14^ map into the linker regions that connect the catalytic and Bateman domains and are directly involved in the stabilization of the different conformations that the hinge bending residues adopt. This observation is indicative of the potential physiological relevance of the IMPDH conformational switch.

Altogether, our results do not only contribute new knowledge on the regulation of IMPDHs but also expand the repertoire of molecular mechanisms by which Bateman domains modulate the activity of the associated enzymes. To this respect, to our best knowledge, this is the first report of a Bateman domain that binds either guanine or adenine nucleotides, triggering different responses to regulate the activity of the associated enzyme. Moreover, our results pave the road to the development of molecules that target the conformational switch to modulate the function of IMPDH. Given the relevance of IMPDH, these molecules might provide new approaches to combat retinopathies, as well as novel strategies to target IMPDHs and, therefore, inhibit cell proliferation.

## Results

### The Bateman domains of AgIMPDH bind both adenine and guanine nucleotides

**Figure 1:**
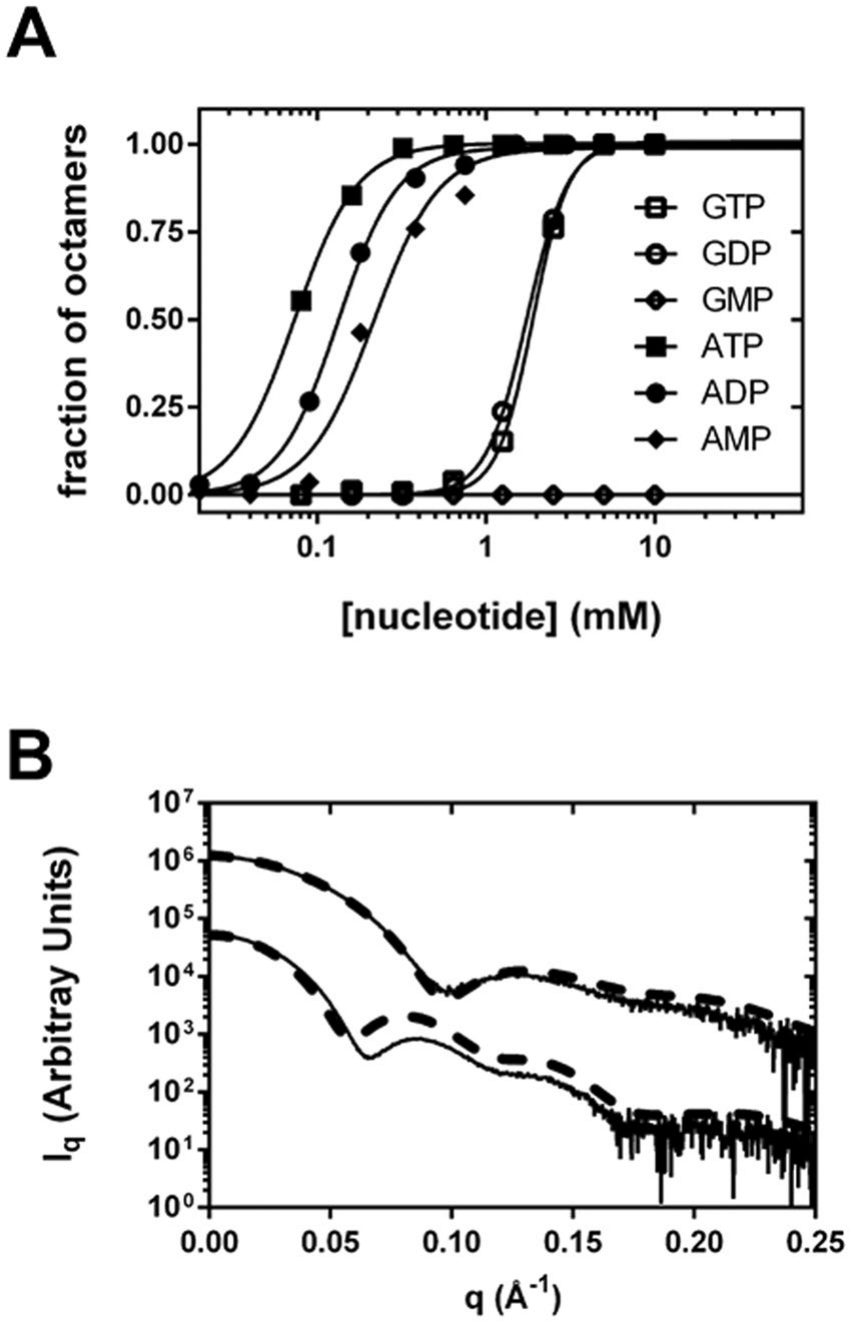
Guanine and adenine nucleotides induce octamers of AgIMPDH with different conformations. (**A**) Fractions of octamers/tetramers of AgIMPDH in solution (4 mg mL^−1^) at different nucleotide concentrations, as determined by SAXS experiments. (**B**) SAXS profiles of AgIMPDH in in the presence of 5 mM GDP (upper continuos line) and 3 mM ATP (botton continuos line). Identical results were obtained for GTP and GDP, as well as for ATP, ADP and AMP (not all curves are shown here to facilitate visualization). The thick dashed lines show the theoretical SAXS profiles calculated from the crystallographic structures fitted to the experimental ones. The SAXS profiles have been conveniently displaced along the y-axis to facilitate visualization.

We have recently reported that GTP/GDP bind to the regulatory Bateman domain of human and fungal IMPDHs and allosterically inhibit their catalytic activity. In contrast, ATP did not show a significant effect on catalytic activity^5^. Nonetheless, given that multiple examples of Bateman domains that bind adenine nucleotides have been reported^10–13^ and following recent results showing the binding of ATP to prokaryotic IMPDHs^4, 24, 26^, we tested whether this was also the case for eukaryotic IMPDHs. We found that ATP, ADP and AMP readily bound to AgIMPDH inducing octamers with K_1/2_ in the low micromolar range (Fig. 1A), despite these nucleotides showed only very slight activation effects on AgIMPDH catalytic activity (Supplementary Fig. 2A). These octamers can also be induced by purine nucleotides at low protein concentrations, such as those in the range used for the enzymatic activity assays (Supplementary Fig. 2B). As expected, neither adenine (ATP/ADP/AMP) nor guanine (GTP/GDP) nucleotides were able to affect the enzymatic activity (Supplementary Fig. 2A) or the oligomerization state (Supplementary Fig. 2C) of a mutant that lacks the Bateman domain (AgIMPDH-ΔBateman) or a point-mutant that cannot be inhibited allosterically by guanine nucleotides (AgIMPDH-R226P^5^). Therefore, these data demonstrate that the adenine and guanine nucleotide binding sites are found within the regulatory Bateman domain of IMPDH.

### Adenine nucleotides induce stretched octamers where the finger domains do not interact

We next studied by Small Angle X-ray Scattering (SAXS) the overall shape and the changes in the structure of AgIMPDH induced by adenine and guanine nucleotides. Interestingly, the binding of ATP/ADP/AMP induced octamers remarkably different from those obtained with GTP/GDP (Fig. 1B). Therefore, the comparison of the full-length structures in the active (ATP/ADP/AMP-induced) and inhibited (GTP/GDP-induced) conformation define a nucleotide-controlled molecular switch of AgIMPDH. To elucidate the details of the differences between ATP and GTP-induced octamers, we solved the structure of AgIMPDH co-crystallized with ATP at 2.4 Å resolution. The crystal belonged to the space group P2_1_ (Table 1) and contains a complete octamer in the asymmetric unit. The eight monomers of the octamer were overall identical in structure, with the highest r.m.s.d. difference being 0.8 Å between chains A and E. The electron density for one of the tetramers (chains E, F, G and H) was less defined that the in the other tetramer (chains A, B, C and D). The octamer found in the crystal reliably represents the octamers found in solution, as demonstrated by the reasonable agreement between the SAXS and the crystallographic data (Fig. 1B). The main difference observed between the theoretical profile (derived from the crystallographic structure) and the experimental one is the displacement of the peak with the maximum at about 0.085 Å^−1^ (experimental profile) to around 0.080 Å^−1^ (theoretical profile), which might indicate that the crystal lattice induces a slight compaction of the octamers with respect to those found in solution. As expected, the maximum of this peak is further displaced in the GDP-induced octamers (about 0.13 Å^−1^; Fig. 1B), indicating that these octamers are significantly more compact than the ATP-induced ones.

**Table 1:** X-ray crystallography data collection and refinement statistics.

	AgIMPDH-ATP	AgIMPDH_ATP/GDP
**Data collection**		
Space group	P2_1_	I4
**Cell dimensions**		
*a, b, c* (Å)	127.89, 152.09, 152.26	147.88, 147.88, 103.54
*α, β, γ* (°)	90.00, 93.03, 90.00	90.00, 90.00, 90.00
Resolution (Å)	127.7–2.40 (2.49–2.40)	46.76–2.46 (2.55–2.46)
*R*_*merge*_	0.21 (1.36)	0.14 (1.44)
*I/σI*	5.8 (1.3)	10.64 (1.4)
Completeness (%)	99.8 (99.3)	99.78 (99.3)
Redundancy	6.5 (6.3)	13.1 (13.5)
**Refinement**		
Resolution (Å)	2.4	2.5
No. reflections	226311 (22460)	40435 (4028)
R-work	0.2511 (0.3171)	0.2270 (0.4035)
R-free	0.2729 (0.3421)	0.2462 (0.4292)
No. atoms		
Protein	22502	7218
Ligand/ion	752	234
Water	1277	51
**B-factors**		
Protein	71.39	75.80
Ligand/ion	81.58	68.76
Water	48.66	59.40
**R.m.s deviations**		
Bond lengths (Å)	0.015	0.014
Bond angles (°)	1.68	1.26

Statistics for the highest-resolution shell are shown in parentheses. Friedel mates were averaged when calculating reflection statistics. Data for both structures were collected using a single crystal.

The Bateman domains were clearly visible for all eight monomers, and each of them contained two ATP molecules, as well as a Mg^+2^ ion, bound to the nucleotide canonical binding sites (Supplementary Fig. 3). All bound nucleotides were refined with occupancy 1.00, except for some of the ATP molecules in chains G and H (ATP603G, ATP602H and ATP603H that were refined with occupancies 0.76, 0.91 and 0.80, respectively). There was no clear electron density for a significant part of the finger domains, suggesting that they are mobile within these octamers. Unexpectedly, a third ATP molecule was bound in the catalytic domain, in the adenosine sub-site of the NAD^+^ pocket, within the active site (Supplementary Fig. 4). This ATP molecule adopted a similar conformation as NAD^+^ and analogs -such as tiazofurin- bound to human IMP dehydrogenases^27^.

**Figure 2:**
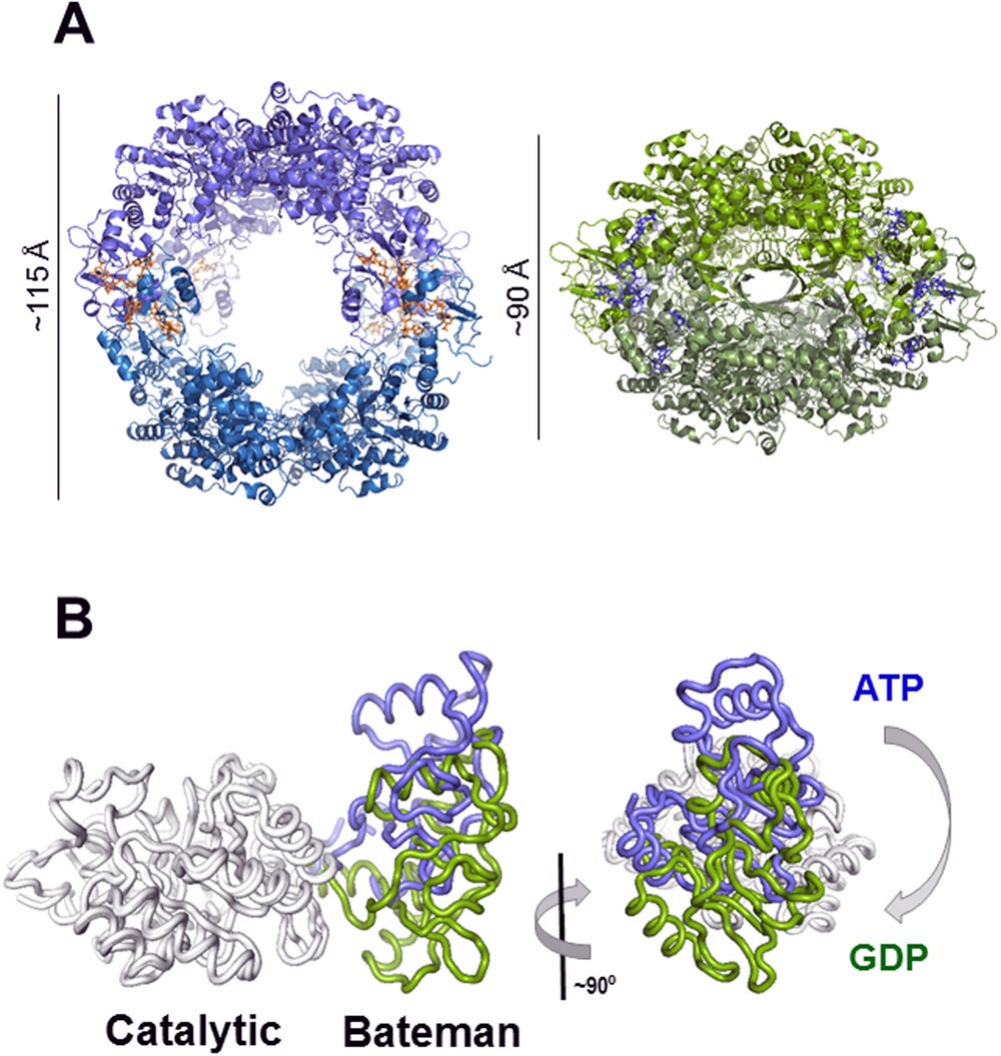
The conformational switch of AgIMPDH. (**A**) High-resolution structures of AgIMPDH in complex with ATP (left panel in blue cartoons) and GDP (right panel in green cartoons^5^). ATP and GDP molecules are shown in orange and blue sticks, respectively. (**B**) Ribbon representation of monomers of AgIMPDH bound to ATP (blue) or GDP (green), with the catalytic domain superimposed.

As expected from our SAXS results (Fig. 1B), the conformation of the ATP-induced octamers significantly differed from the GDP-induced one; the later were compacted along the four-fold symmetry axis and forced the finger domains of the upper and lower tetramer to interact. In contrast, the former were stretched out along the four-fold symmetry axis, allowing the fingers to move freely in the bulk solvent (Fig. 2A). The overall structure of the isolated catalytic and Bateman domains remained unchanged, i.e. they behaved basically as rigid bodies, though their relative orientation notably differed: upon GDP binding, the Bateman domain rotated ~24° and translated ~10 Å with respect to the catalytic domain (Fig. 2B). This change was the consequence of the large differences in the structure of the linker regions, where the sequences Glu117-Ala123 and Lys231-Pro236 were identified as the hinge bending residues (Supplementary Fig. 5A) that orchestrate the conformational change. Remarkably, the hinge bending residues were close to and/or directly interacted with the bound nucleotides (Supplementary Fig. 5B), readily explaining how guanine and adenine nucleotides stabilize the different conformations of the hinges, thereby inducing changes in the structure of AgIMPDH. Interestingly, several residues in AgIMPDH that correspond to missense mutations in HsIMPDH1 associated to retinopathies^14^ lie within the linker regions and/or are directly involved in stabilizing the different conformations of the hinge bending residues (Supplementary Fig. 5), pointing to an important physiological role of the IMPDH conformational switch within cells.

In the octamers, the changes within the monomers were accompanied by alterations in the interface of the interacting pairs of Bateman domains: upon GDP binding, the bending angle was ~30° more closed than in the ATP complex (Supplementary Fig. 6). As a result, the GDP-bound inhibited octamers were compacted along the four-fold symmetry axis with respect to the ATP-induced active octamers. Thus, our structural data further support our previously proposed hypothesis that the compaction of the octamers forces the interaction of the finger domains that results in the subsequent catalytic activity inhibition by altering the internal dynamics of the catalytic site and decreasing the binding affinity and Km values of the substrate IMP^5^.

### Adenine and guanine nucleotides compete for the canonical sites of Bateman domains

In the crystal structure of AgIMPDH-ATP, electron density was unequivocally assigned to two ATP molecules and one Mg^+2^ ion bound to the nucleotide canonical sites of each of the Bateman domains of the AgIMPDH octamer (Supplementary Fig. 3). For the sake of clarity, from now on, we will name ATP1/GDP1 and ATP2/GDP2 to the nucleotides bound into the first and second canonical sites of archetypal Bateman domains^11^, and GDP3 to the nucleotide bound into the non-canonical binding site described in the AgIMPDH-GDP structure^5^.

**Figure 3:**
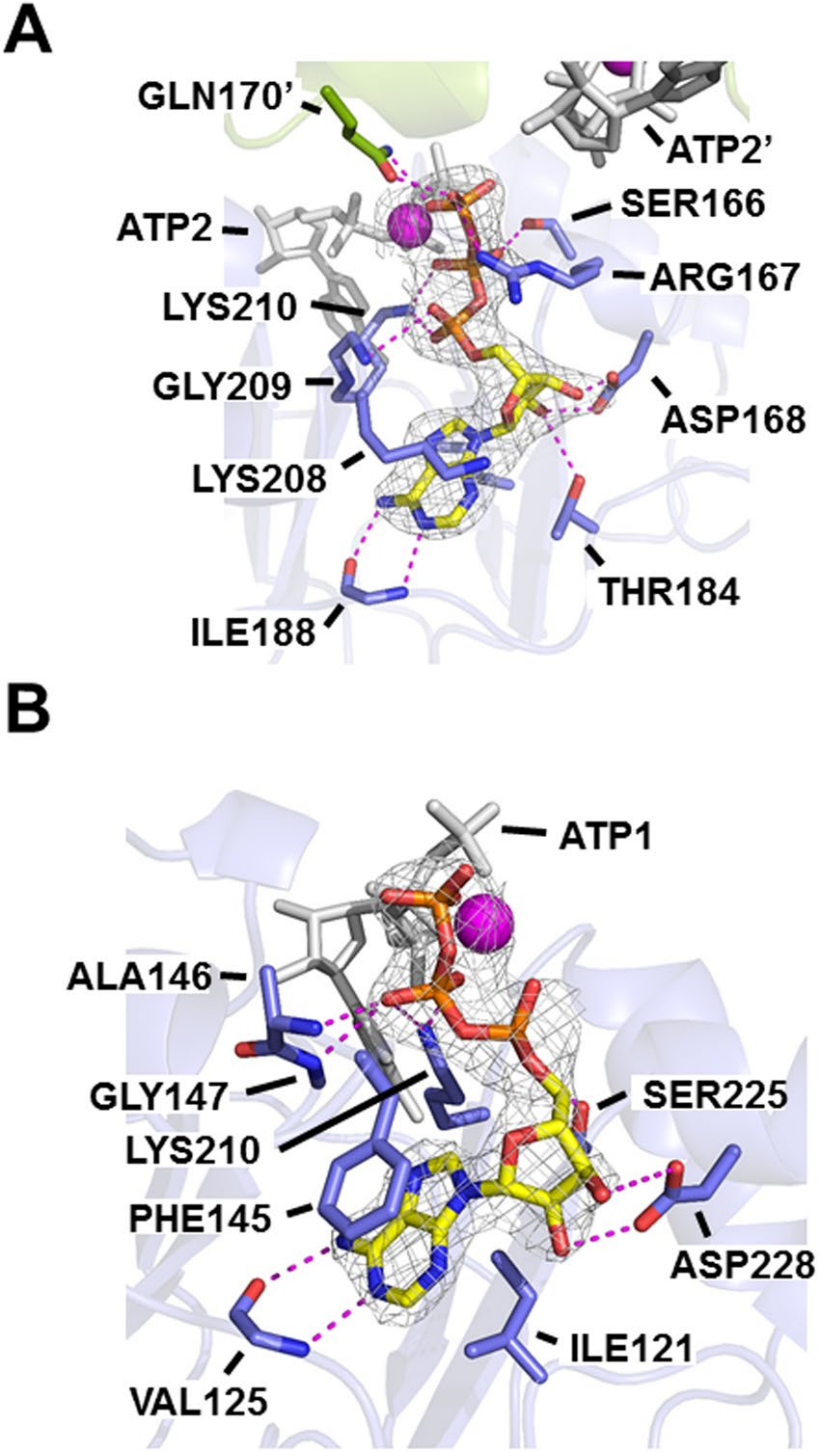
The Bateman domain of AgIMPDH binds two ATP molecules. Close-up view of the ATP1 (**A**) and ATP2 (**B**) bound to the canonical nucleotide binding sites of the Bateman domain of AgIMPDH. AgIMPDH protein is represented in blue semi-transparent cartoons with key interacting residues and ATP molecules shown in sticks. The Mg^+2^ ion is shown as a magenta sphere. In (**A**) the adjacent monomer and the side chain of residue Gln170’ are shown in green cartoon and sticks, respectively. Key interactions are represented as magenta dashes. The grey mesh around the nucleotides and the Mg^+2^ ion represents the ommit 2mF_o_ − DF_c_ electron density map contoured at the 1σ level.

In the AgIMPDH-ATP complex, both ATP1 and ATP2 adopted an extended conformation that coordinates a Mg^+2^ ion between their β- and γ-phosphate groups (Supplementary Fig. 3). The recognition of the adenine rings was mostly mediated by hydrogen bonds from the backbone atoms of residues Ile188 and Val125 to the N1 and N6 nitrogen atoms of the adenine ring of ATP1 and ATP2, respectively. The nucleobases were sandwiched by the side chains of residues Lys208 and Ile163 for ATP1, and residues Phe145 and Ile121 for ATP2 (Fig. 3). The hydroxyl groups of the ribose moiety tightly interacted with the side chains of the fully-conserved Asp168 and Asp228, which constitute an archetypal sequence signature of CBS motifs^11^. Finally, several residues coordinated the phosphates mostly by ionic interactions: Ser166, Arg167, Gly209 for ATP1, and Ala146, Gly147 for ATP2. Additionally, the primary amine of the side chain of Lys210 simultaneously coordinated the phosphates of both ATP1 and ATP2 (Fig. 3).

### GDP3 staples the catalytic and Bateman domains into a fixed conformation

**Figure 4:**
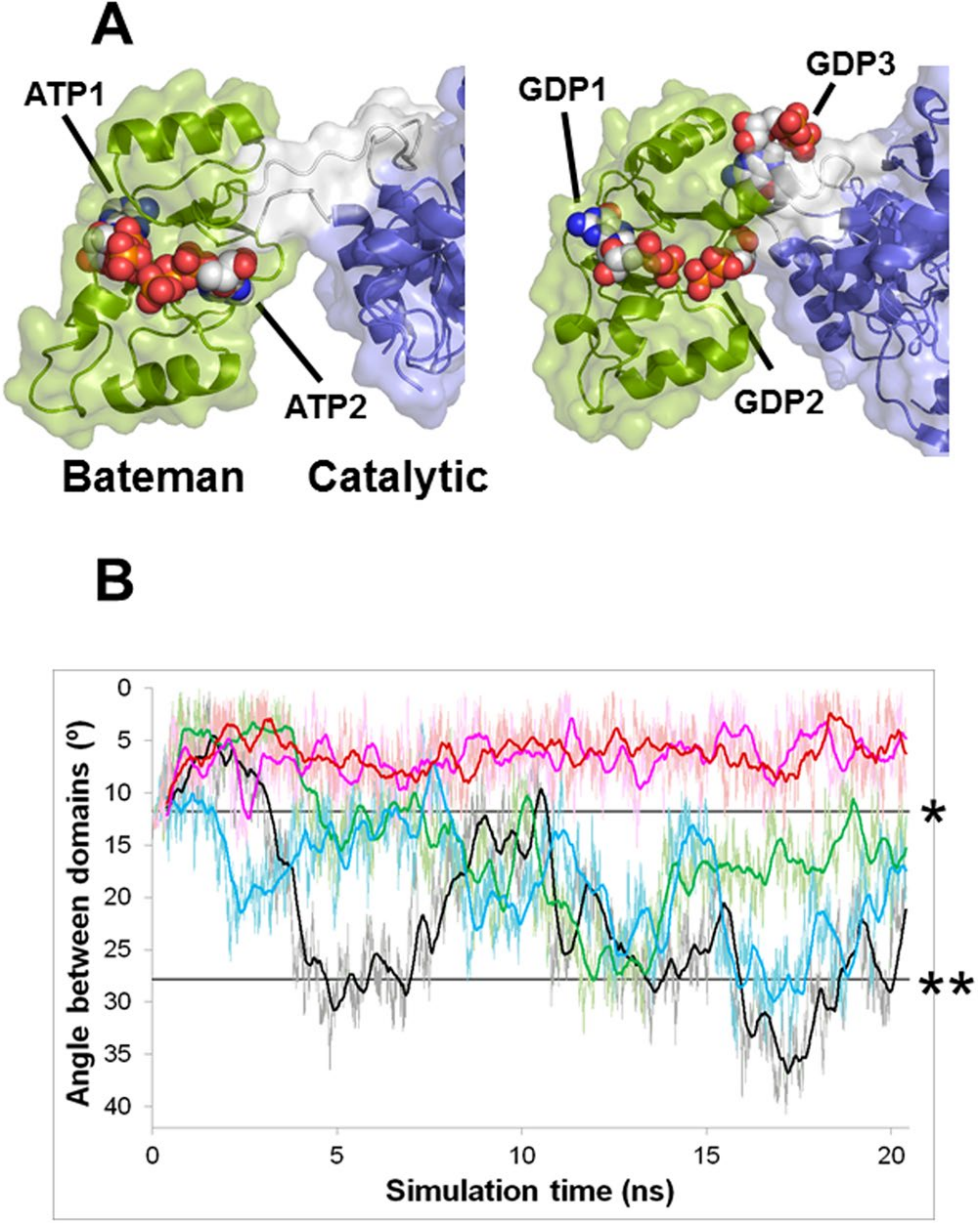
GDP2 and GDP3 staple the catalytic and Bateman domains into a fixed inhibited conformation. (**A**) Cartoon and surface representation of a monomer of AgIMPDH-ATP (left) and AgIMPDH-GDP (right). Nucleotide atoms are shown as spheres. The catalytic domain is coloured in blue, the Bateman domain in green and the hinge bending residues (linkers) in grey. (**B**) Angle formed between the mass centers of the catalytic, linker and Bateman domains of a monomer of AgIMPDH bound to different nucleotides during the molecular dynamics computer simulations. Black: APO, blue: GDP1/GDP2, green: ATP1/ATP2, pink ATP1/GDP2/GDP3, and brown: GDP1/GDP2/GDP3. The thin lines show the raw data and the smoother thick lines are the mobile mean of the angle along 20 structures. The straight black lines marked with one or two asterisks indicate the angles formed in the structures of AgIMPDH-GDP (starting structure for all simulations) and AgIMPDH-ATP, respectively.

Notable differences were observed for the binding of adenine and guanine nucleotides to AgIMPDH, particularly for ATP2 and GDP2 that adopted completely different conformations within the second canonical nucleotide binding site (Supplementary Fig. 7A). In addition, GDP2 and GDP3 extensively contacted both catalytic and Bateman domains, as well as the linkers connecting them, which act as hinge bending residues. In contrast, ATP2 did not interact with either the catalytic domain or the linker regions (Fig. 4A). These observations suggest that ATP is inducing an active conformation of AgIMPDH that would somehow allow certain flexibility between the catalytic and Bateman domains. In contrast, the binding of GDP seems to staple the catalytic and Bateman domains into a fixed conformation that seriously compromises catalytic activity.

To further investigate this hypothesis, we performed computational Molecular Dynamics (MD) simulations of monomers of AgIMPDH bound to different nucleotides. Remarkably, the simulations showed that the inhibited conformation observed in the AgIMPDH-GDP complex was strongly stabilized when GDP2 and GDP3 were bound to their respective sites (Fig. 4B). In contrast, when ATP was bound to the two canonical sites, AgIMPDH could oscillate between the active (ATP bound) and inhibited (GDP bound) conformations (Fig. 4B). Similarly, AgIMPDH monomers with GDP1 and GDP2 bound to the two nucleotide canonical binding sites (and the third non-canonical site empty), or without any nucleotide bound, oscillated between the active and inhibited conformations (Fig. 4B). We then performed Steered Molecular Dynamics simulations (SMD), that employ a pulling force to drive a structure into a different conformation during a MD simulation^28^. SMD simulations clearly showed that the system could be easily driven from the inhibited to the active conformation when there was no guanine nucleotide bound to the non-canonical site. In marked contrast, an increasing supply of energy (accumulated work) was needed to exit from the inhibited conformation when this site was occupied by GDP (Supplementary Fig. 8).

Altogether, these data suggest that the binding of GDP3 strongly stabilizes a conformation that staples the catalytic and Bateman domains, inducing the compaction of the AgIMPDH octamer along the four-fold symmetry axis.

### GTP and GDP inhibit ATP-induced AgIMPDH octamers

**Figure 5:**
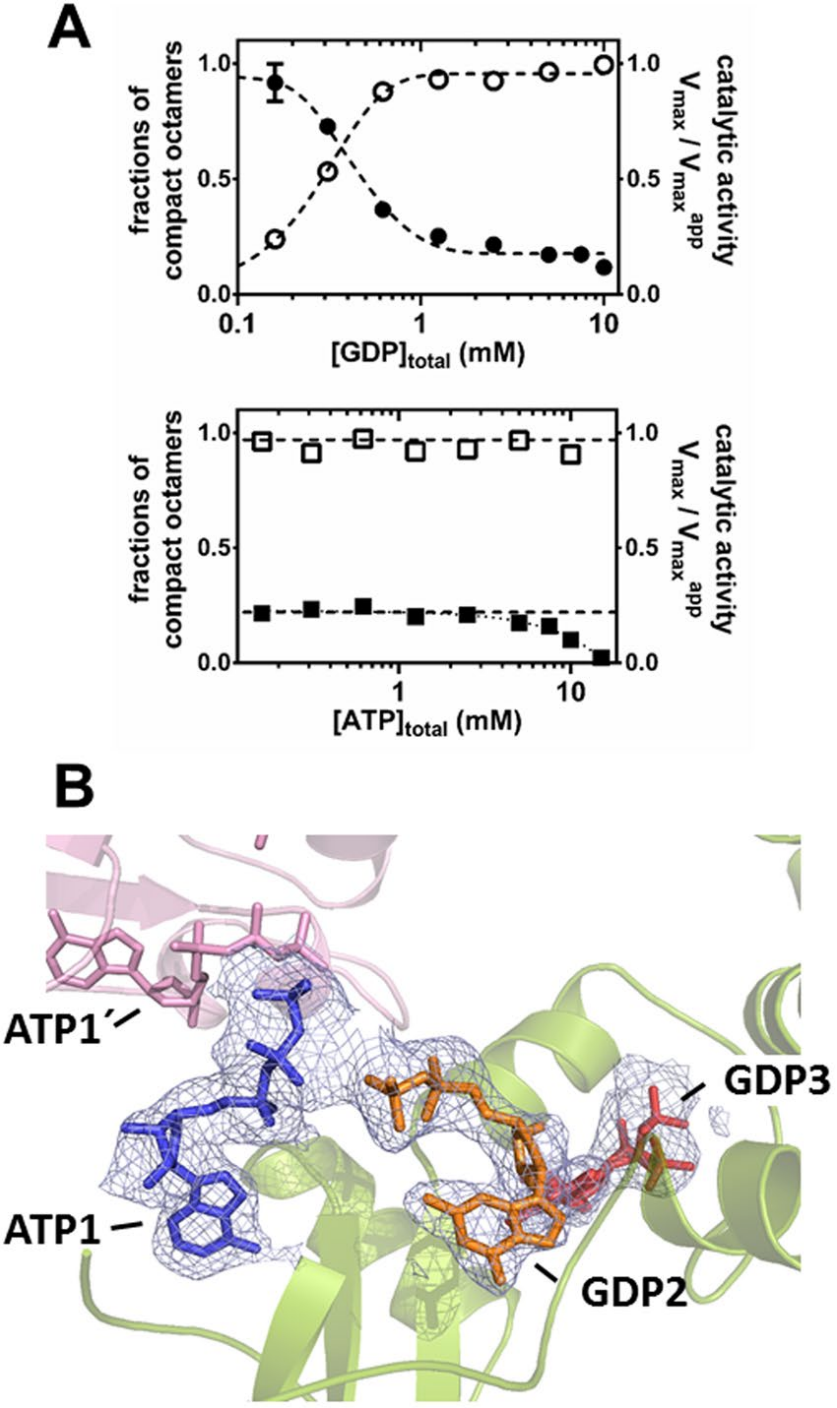
The conformational switch of AgIMPDH is unidirectional. (**A**) Plots representing the fraction of compact octamers (empty symbols) and the catalytic activity (filled symbols) *versus* nucleotide concentration. Upper panel: 3 mM ATP fixed *versus* increasing concentrations of GDP. Lower panel: 5 mM GDP fixed *versus* increasing concentrations of ATP. Data points represent the mean value and the standard errors. At the fixed concentrations of ATP (3 mM) and GDP (5 mM) used for these experiments, all AgIMPDH remained as octamers with no detectable fraction of tetramers, as determined by SAXS. (**B**) Close-up view of the nucleotides bound to the Bateman domain in the structure AgIMPDH-ATP/GDP. The protein is represented in green cartoons and the nucleotides in coloured sticks (ATP1 in blue, GDP2 in orange, and GDP3 in red). The interacting Bateman domain form the other tetramer is shown in light pink. The grey mesh around the nucleotides represents the ommit 2mF_o_ − DF_c_ electron density map contoured at the 1σ level.

The data presented above clearly demonstrate that ATP and GDP compete for the canonical nucleotide binding sites of the Bateman domain. However, the intracellular ATP concentrations are usually higher than those of GTP/GDP^29^ and, moreover, ATP induces AgIMPDH octamers more efficiently than GTP/GDP (Fig. 1A), which challenges the efficacy of GTP/GDP inhibition *in vivo*. Therefore, we decided to study the catalytic activity of AgIMPDH *in vitro* when both nucleotides are present in the solution. The upper panel of Fig. 5A clearly shows that GDP was able to inhibit the active octamers induced by ATP. Remarkably, the GDP-induced inhibition of the ATP-active octamers was fully correlated to the compaction of the octamers, as demonstrated by SAXS experiments (Fig. 5A, upper panel). In contrast, ATP could not activate the inhibited octamers induced by GDP (Fig. 5A, lower panel). At the highest concentrations, ATP induced a further decrease of the catalytic activity, most probably by competing with NAD^+^ for the adenine pocket within the active site. This hypothesis is well supported by the finding of an ATP molecule bound to the adenine subsite of NAD^+^ in our AgIMPDH-ATP crystallographic structure (Supplementary Fig. 4). Nevertheless, we do not expect this mechanism to be physiologically relevant, given the high ATP concentrations required (Fig. 5A).

We further investigated these findings by solving the structure of AgIMPDH co-crystallized with ATP and GDP at 2.5 Å resolution (Table 1). The overall structure of AgIMPDH-ATP/GDP was essentially identical to that of GDP octamers with only small variations (Supplementary Fig. 7B), which were most probably due to differences in the crystal packing. Interestingly, the structure showed ATP bound to the first canonical site and GDP bound to the second and third nucleotide binding sites (Fig. 5B). The binding modes of ATP1 and GDP2/GDP3 in this structure were essentially identical to the binding modes observed for these nucleotides in their respective ATP and GDP complex structures. Also, as expected, the AgIMPDH monomers bound to ATP1 and GDP2/GDP3 behaved essentially identical than the AgIMPDH-GDP1/GDP2/GDP3 complex in MD and SMD computational simulations (Fig. 4B and Supplementary Fig. 8). Therefore, the occupancy of the second canonical and the third non-canonical nucleotide binding sites of the Bateman domain determined the conformation of AgIMPDH octamers and, therefore, its catalytic activity. Remarkably, these sites are the most divergent between eukaryotic and prokaryotic IMPDHs, in agreement with the finding that the allosteric regulation is differentially controlled between eukaryotic and prokaryotic organisms^5^.

## Discussion

Bateman domains are conserved structural modules, present in a large diversity of functionally unrelated proteins. They regulate a variety of functions in response to the cellular energy charge, being involved in transport on Mg^+2^ ions, osmoregulation, chloride ion channel regulation, nitrate transport or in the pyrophosphatase activity^11^. Nonetheless, despite the demonstrated relevance of Bateman domains in the modulation of diverse protein functions, the molecular mechanisms underlying regulation have only just begun to emerge. This is mostly due to the challenge to obtain pairs of full-length structures of active and inactive protein complexes^10, 11^. In this study, we have determined the high-resolution structures of the full-length enzyme IMPDH from the industrial fungus *Ashbya gossypii* in complex with ATP, forming active octamers, and with ATP/GDP, forming octamers with compromised catalytic activity. The comparison of both structures allowed us to decipher the molecular details of a novel regulatory mechanism mediated by Bateman domains that respond differently to adenine or guanine nucleotides. These nucleotides control a conformational switch that allosterically regulates the catalytic activity of IMPDH. To our best knowledge, this is the first description of a Bateman domain that binds adenine and/or guanine nucleotides and triggers differential signals depending on the nucleotide bound. The exact role of the described conformational switch within cells must be further investigated, but the observation that several missense mutations in HsIMPDH1 associated to human retinopathies lie within the hinges is indicative of an important physiological relevance that definitively deserves to be explored.

The results reported here further support our hypothesis that GTP and GDP induce the association of two IMPDH tetramers to form octamers where the finger domains are forced to interact, compromising the catalytic efficiency of the enzyme. The interaction of the finger domains might alter the internal dynamics of the active site, disfavoring substrate binding, as previously demonstrated^5^, and probably also impeding the movement of the catalytic mobile flap, hence, strongly stabilizing some of the various conformations that the active site adopts during the catalytic cycle^3^. Indeed, adenine nucleotides induce octamers where the finger domains do not interact, which explains why these nucleotides do not significantly affect the catalytic activity of AgIMPDH, as comparable dynamics are expected for the finger domains of tetramers and ATP-induced octamers.

The fact that the non-canonical nucleotide binding site of eukaryotic IMPDHs is exclusive of GTP/GDP, implies that ATP cannot compete guanine nucleotides out of this site and allows GTP/GDP-induced allosteric inhibition to proceed independently on the adenine nucleotide concentrations. It also implies that the conformational switch is unidirectional, *i.e*. guanine nucleotides can inhibit ATP-induced octamers, but ATP cannot reverse GDP inhibition. This is especially relevant in physiological terms, given that the intracellular concentrations of ATP are usually much higher than those of guanine nucleotides^29, 30^, unbalancing the competition of guanine and adenine nucleotides for the binding sites in favor of the later. Interestingly, the K_1/2_ value of the GDP-induced inhibition of ATP octamers, that correlates with octamer compaction, are about 0.3–0.4 mM (Fig. 5A) that lie in the range of the physiological concentrations of guanine nucleotides^29, 30^, highlighting the potential physiological relevance of this mechanism of enzymatic activity inhibition.

Remarkably, adenine nucleotides induce AgIMPDH active octamers *in vitro* at concentrations significantly lower than the expected *in vivo*: intracellular ATP concentrations vary from 0.5–10 mM^29^, while the K_1/2_ of octamer induction *in vitro* is around 0.1 mM. This finding points to the possibility that AgIMPDH might exist mainly as ATP-bound octamers within cells. Similarly, *P. aeruginosa* IMPDH enzyme has been proposed to be an octamer *in vivo*, according to crosslinking results^31^. Further experiments are needed to elucidate the oligomeric state of IMPDH within cells.

The conformational switch described herein for AgIMPDH, that we speculate might presumably occur in most other eukaryotic IMPDH enzymes, seems to be also present in bacteria. Indeed, cryo-electron microscopy revealed two types of octameric assemblies for the APO (inhibited) and ATP-bound (active) forms of *Pseudomonas aeruginosa* IMPDH^4^ that closely resemble the inhibited (GDP bound) and active (ATP bound) conformations of AgIMPDH, respectively. It is expected that ATP binding activates the *P. aeruginosa* IMPDH inhibited octamers –in which finger domains interact- by stretching these octamers out and relieving the finger domain interactions. Altogether, prokaryotic and eukaryotic IMPDHs possess a conformational switch that allow them to oscillate between active and inhibited conformations, though the way the switch is controlled by purine nucleotides is different. Thus, with the available data, it is reasonable to speculate that a conformational switch –controlled by ATP- was already present in the common ancestor of eukaryotic and prokaryotic IMPDHs. Along evolution, eukaryotic IMPDHs have likely modified the second canonical binding site and introduced a third one, exclusive for guanine nucleotides, which allowed the enzyme to be allosterically inhibited by GTP and GDP. Why eukaryotic IMPDHs need guanine nucleotides to inhibit their catalytic activity while some prokaryotic IMPDHs remain inhibited until ATP binding activates them remains to be determined, but it represents an excellent example of how divergent evolution has adapted an enzyme to the specific metabolic requirements of each particular organism. Future experiments will be directed to decipher the sequence and structural determinants that define these differences.

In summary, our results describe a novel conformational switch, controlled by purine nucleotides, that allosterically regulates the activity of eukaryotic IMPDHs. The switch is expected to modulate the metabolic flux through the guanine nucleotide biosynthetic pathway in response to high levels of GTP/GDP and/or an imbalance between the guanine and adenine nucleotide pools. Although the exact role *in vivo* of this switch remains to be explored, the finding that pathological mutations map within the hinges, points to an important physiological function that definitively deserves to be explored. Given the therapeutic relevance of IMPDH, our work settles a solid basis for the development of molecules that target the Bateman domain to either up or downregulate the activity of IMPDH. These molecules would provide ways to combat hereditary retinopathies associated with mutations in this domain, as well as new approaches to target IMPDH and, therefore, inhibit cell proliferation. Moreover, our results also suggest the intriguing possibility of using the Bateman domain of eukaryotic IMPDHs as building blocks to design regulatable proteins for synthetic biology and biotechnological applications.

## Methods

### Proteins and nucleotides

Expression and purification of AgIMPDH proteins was performed as previously described^5, 17^. Nucleotides were purchased from Sigma-Aldrich.

### Enzyme kinetics assay

IMPDH activity was assayed at 28 °C using 96 well microtiter plates by monitoring the reduction of NAD^+^ to NADH and the subsequent increase in absorbance at 340 nm, as previously described^17^. AgIMPDH at 20 µg/mL in buffer 100 mM Tris-HCl, 100 mM KCl, 2 mM DTT, pH 8.0 was assayed using 0.5 mM NAD^+^ and 0.019–5 mM IMP as substrates, in the presence of different concentrations of purine nucleotides. The total amount of MgCl_2_ was adjusted for each nucleotide concentration to keep free MgCl_2_ concentration constant at 2 mM. Free MgCl_2_ was calculated from the total MgCl_2_ concentration by solving the multiple equilibria, which take into account the cation binding to all nucleotides (ATP and/or GDP) present in the solution using previously published stability constants Mg^+2^-nucleotide in similar experimental conditions^32, 33^. The experimental data were fitted to the Michaelis-Menten equation using GraphPad Prism (GraphPad Prism Software, Inc.).

### Chemical crosslinking

Protein samples at 2 mg/mL, or 50 µg/mL, were crosslinked at 25 °C for 30 minutes with 1 mM DSS (Pierce, Thermo Fisher Scientific) in buffer 20 mM HEPES, 100 mM KCl, 2 mM MgCl_2_, 1 mM DTT, pH 8.0. Reactions were quenched by an excess of Tris-HCl, pH 8.0 and the samples were analyzed on 4–15% gradient SDS–PAGE (BioRad).

### Small Angle X-ray Scattering (SAXS)

SAXS measurements were performed at the P12 beamline^34^ at EMBL-Hamburg, as described previously^5^. Samples of AgIMPDH at 4 mg/mL in buffer 20 mM TrisHCl, 150 mM KCl, 5 mM MgCl_2_, 3 mM DTT, pH 8.0, were measured in the presence of increasing amounts of nucleotides (total concentration of nucleotides ranging from 0.16 to 10 mM). The fractions of active and inhibited octamers were calculated with the program OLIGOMER that fits an experimental scattering curve from a multicomponent mixture to find the volume fractions of each component in the mixture^35^. The theoretical solution scattering profiles were calculated from the crystal structures and fitted to the experimental data using the program CRYSOL^36^.

### Crystallization and structure determination

Crystals of the complex AgIMPDH-ATP were grown at room temperature using the vapor diffusion method by mixing a protein solution at 10 mg ml^−1^ in 10 mM Tris-HCl, 100 mM KCl, 2 mM DTT, 7.5 mM ATP, 3 mM MgCl_2_, pH 8.0, with an equal volume of mother liquor consisting of 0.02 M D-Glucose, 0.02 M D-Mannose, 0.02 M D-Galactose, 0.02 M L-Sucrose, 0.02 M D-Xylose, 0.02 M N-Acetyl-D-Glucosamine, 0.05 M Imidazole, 0.05 M Mes, pH 6.5, 20% (w/v) Glycerol, 20% (w/v) PEG-4000. Protein crystals were flashed-cooled in liquid nitrogen and data were collected at 100 K, using monochromatic X-rays of 0.9999 Å wavelength, at the beamlines I03 and XALOC^37^ of the Diamond Light Source synchrotron (UK) and ALBA synchrotron (Spain), respectively. Intensities were indexed using methods for multi-lattice diffraction data implemented in the software DIALS^38^.

Crystals of the complex AgIMPDH-ATP/GDP were grown identically (same buffer and protein concentration) in the presence of 3 mM mM ATP and 6 mM GDP in mother liquor consisting of 0.1 M Lithium Acetate, 0.1 M BisTris pH 6.0, 20% (w/v) Sokolan-CP42. Crystals were immersed in NVH oil for cryoprotection before being flashed-cooled in liquid nitrogen and data were collected as described above for AgIMPDH-ATP. Diffraction intensities were indexed and integrated by using the software XDS and scaled with XSCALE^39^.

Data were phased by molecular replacement using the program PHASER^40^ within the CCP4 suite^41^, using as templates the isolated structures of the catalytic and Bateman domains of the complex AgIMPDH-GDP^5^. The structures were refined using the PHENIX crystallographic software package^42^, alternating manual modelling with COOT^43^. Rigid body, gradient-driven positional, restrained individual isotropic B-factor and TLS^44^ were used for structure refinement.

### Structural analyses

Protein domain motion analyses were performed with DynDom^45^, that determined the hinge axis and the hinge bending residues, by comparing the two conformations observed in the structures of AgIMPDH-ATP and AgIMPDH-ATP/GDP or AgIMPDH-GDP^5^. The three dimensional structure figures were done with the software PyMOL (The PyMOL Molecular Graphics System, Version 1.8 Schrödinger, LLC).

### Computer Molecular Dynamics simulations

Free molecular dynamics (MD) simulations of protein monomers were performed using the AMBER 14 MD package^46^. The 3D structures were solvated using the LEaP module of AMBER, being 12 Å the closest distance between any atom of the protein and the periodic box boundaries. Na^+^ counter-ions were added to neutralize the charge of the systems. All free MD simulations were performed in the NPT (constant temperature, constant pressure) ensemble, using the PMEMD program of AMBER and the parm99 force field^46^. SHAKE algorithm was used allowing a time step of 2 fs. AgIMPDH-GDP monomer was used as reference in all cases, substituting the nucleotides by ATP or void positions in the different simulated conditions. The systems were initially relaxed with 15,000 steps of energy minimization with a cut-off of 12 Å and MD simulations were started with a 20 ps heating phase. During minimization and heating, the Cα trace dihedrals were restrained with a force constant of 500 kcal mol^−1^ rad^−2^. The constrains were maintained in the Bateman and catalytic domains while in the inter-domain contacting link (residues 98–127 and 225–251 respectively) they were gradually released in an equilibration phase in which force constant was gradually reduced to 0 along 200 ps. After the equilibration phase, 20 ns productive MD simulations were obtained for all the systems, monitoring the angle between the centers of mass of the Bateman and the catalytic domain in all computation steps.

In order to compare the work necessary to separate the Bateman and catalytic domains from the AgIMPDH-GDP position to the AgIMPDH-ATP position, calculation of the accumulated work (kcal mol^−1^) was performed for each case using steered Molecular Dynamics (SMD). During each SMD trajectory the distance between two residues located in each domain (Cα of Cys249 and Phe143, respectively) was forced to separate from the initial 19,1 Å (representative of the GDP1/GDP2/GDP3 condition) to a final distance of 27.7 Å (representative of the ATP1/ATP2 condition) at constant velocity (2.5 Å ns^−1^) with a spring constant of 5 kcal mol^−1^ Å^−2^ (Supplementary Fig. 8), in the range of conditions previously used in similar SMD studies^47, 48^. For each calculation step, the distance between the residues was recorded to later reconstruct the forces and works generated along each trajectory.

## Electronic supplementary material

Supplementary Information
