# Supplementary material for: A nucleotide-controlled conformational switch modulates the activity of eukaryotic IMP dehydrogenases
Source: Sci Rep. 2017 Jun 1;7:2648. doi: 10.1038/s41598-017-02805-x (PMC5454003; doi:10.1038/s41598-017-02805-x)

SUPPLEMENTARY INFORMATION

A nucleotide-controlled conformational switch modulates the activity of eukaryotic IMP dehydrogenases

Rubén M. Buey[1*], David Fernández-Justel[1], Íñigo Marcos-Alcalde[2], Graeme Winter[3], Paulino Gómez-Puertas[2], José María de Pereda[4], and José Luis Revuelta[1*]

[1]Metabolic Engineering Group, Dpto. Microbiología y Genética. Universidad de Salamanca. Campus Miguel de Unamuno, 37007, Salamanca, Spain

[2]Molecular Modelling Group, Centro de Biología Molecular "Severo Ochoa" (CSIC-UAM), ES-28049 Madrid, Spain

[3]Diamond Light Source, Harwell Science and Innovation Campus, Didcot, Oxfordshire OX11 0DE, England

[4]Instituto de Biología Molecular y Celular del Cáncer (ICSIC-Universidad de Salamanca). Campus Miguel de Unamuno, 37007, Salamanca. Spain

*Correspondence: J.L.R. (revuelta@usal.es) and/or R.M.B. (ruben.martinez@usal.es)

SUPPLEMENTARY FIGURE LEGENDS

Supplementary Figure 1. *Structural organization of IMP dehydrogenase*. Upper panel. Multiple sequence alignment of IMPDH from selected organisms. The structural and functional domains are indicated below the sequence alignment, as well as the linker regions that were identified in this work to act as the hinges that allow to switch between the diferent conformations. Lower panel. Cartoon representation of a monomer of IMPDH with the different structural and functional domains indicated. The color code is the same as in the upper panel. The catalytic flap, invisible in the crystal structures of AgIMPDH, is shown as a dashed black line.

Supplementary Figure 2. *The Bateman domain of AgIMPDH binds adenine and guanine nucleotides*. A. Values of V_{max}^{app} / V_{max} at 3 mM (total concentration) of the indicated nucleotides for AgIMPDH-WT, AgIMPDH-ΔBateman and AgIMPDH-R226P. Enzyme concentration used was 20 µg/mL in all the cases. The V_{max} values in the absence of nucleotide were 44.83 ± 2.3, 43.71 ± 4.5 and 39.99 ± 3.6 Abs_{340nm} / second for AgIMPDH-WT, AgIMPDH-ΔBateman and AgIMPDH-R226P, respectively. B. BlueSafe (Nzytech) stained SDS-PAGE of 50 µg/mL cross-linked AgIMPDH-WT in the presence of 3 mM of the indicated nucleotides (total concentration). C. Coomassie Blue stained SDS-PAGE of 2 mg/mL cross-linked AgIMPDH-WT, AgIMPDH-ΔBateman and AgIMPDH-R226P in the presence of 3 mM of the indicated nucleotides (total concentration). The single asterisk indicates octamers and the double asterisk indicates tetramers. The results with ADP and AMP were essentially identical to ATP.

Supplementary Figure 3. *Two molecules of ATP bind to the canonical nucleotide binding sites of the Bateman domain of AgIMPDH.* Stereo view of two interacting Bateman domain (green and blue semi-transparent cartoons) of the AgIMPDH-ATP octamer. Nucleotides are shown in orange sticks and the Mg^{+2} ions are shown as magenta spheres. The dark red mesh around the nucleotides represents the ommit $2mF_o$ - DF_c electron density map contoured at the 1σ level.

Supplementary Figure 4. *ATP bound to the adenosine subsite of NAD^+ in the active site*. A. Cartoon representation of a region of the active site of AgIMPDH showing an ATP molecule bound into the adenosine subsite of NAD^+. The nucleotide and key interacting protein residues are shown in sticks. Some key interations are shown as magenta dashed lines. A glutamine residue from the adjacent monomer (Gln176'; in green) coordinates the ribose hydroxils of ATP. The grey mesh around the nucleotides represents the ommit $2mF_o$ - DF_c electron density map contoured at the 1σ level. B. Superposition of the structures of the unespecifically bound ATP (yellow sticks) of

AgIMPDH-ATP and the NAD^+ analogue selenazole-4-carboxamide adenine dinucleotide (dark red sticks) bound to HsIMPDH2 (PDB code 1b3o).

Supplementary Figure 5. *The mechanical hinges of the AgIMPDH conformational switch.* A. Bending region analysis performed by the software DynDom to determine the rotational transitions at the bending segments. The plots show the changes in the psi-dihedral angles for the hinge bending residues between the conformations observed in the structures of AgIMPDH-ATP and AgIMPDH-GDP. Residues that correspond to missense mutations of HsIMPDH1 associated to retinopathies are marked with an asterisk. Residues Asn122 and Gln233 in AgIMPDH correspond to Thr116 and Arg231in HsIMPDH1, respectively. B and C. Cartoon representation of a monomer of AgIMPDH-ATP (B) and AgIMPDH-GDP (C). The catalytic, Bateman and linker domains are coloured in blue, green and orange, respectively. The nucleotides and the side chain of essential residues to stabilize the conformation of the linker are shown in sticks. Key interactions are represented as yellow dashes. Residues that correspond to missense mutations of HsIMPDH1 associated to retinopathies are marked with an asterisk. Residues Asn122, Asp228 and Gln233 in AgIMPDH correspond to Thr116, Asp226 and Arg231in HsIMPDH1, respectively.

Supplementary Figure 6. *The conformational switch of AgIMPDH.* Cartoon representation of two monomers (one from the upper and the other from the lower tetramers) of the AgIMPDH-ATP (active state; upper panel) and AgIMPDH-GDP (inhibited state; lower panel) octamers. ATP and GDP nucleotides are shown in orange and blue sticks, respectively. The Bateman domains of AgIMPDH-GDP (upper panel) and AgIMPDH-ATP (lower panel) are shown in white cartoons to facilite a side-by-side comparison of both structures. The dashed lines indicate the approximate angle formed by a line that passes parallel to the two beta sheets of the CBS motifs in each Bateman domain.

Supplementary Figure 7. *ATP2 and GDP2 exploit the nucleotide binding sites in the Bateman domain of AgIMPDH in different ways.* A. Close-up view of a structural superimposition of the Bateman domain of AgIMPDH-ATP (blue semi-transparent cartoons), AgIMPDH-ATP/GDP (red semi-transparent cartoons) and AgIMPDH-GDP (green semi-transparent cartoons). Bound nucleotides are shown sticks with the respective colours. B. Cartoon representation of AgIMPDH octamers with ATP bound to the two canonical sites (left panel, in blue cartoons: ATP1/ATP2), ATP bound to the first canonical site and GDP bound to the second canonical and to the non-canonical site (middle panel, in red cartoons: ATP1/GDP2/GDP3) and GDP bound to the two canonical and the non-canonical sites (right panel, in green cartoons: GDP1/GDP2/GDP3).

Supplementary Figure 8. *GDP2 and GDP3 staple the catalytic and Bateman domains into a fixed inhibited conformation.* Accumulated work (kcal mol^{-1}) along the steered molecular dynamics simulations of monomers of AgIMPDH-GDP forced to adopt the AgIMPDH-ATP conformation, when bound to different nucleotides. Black: APO, blue: GDP1/GDP2, green: ATP1/ATP2, pink ATP1/GDP2/GDP3, and brown: GDP1/GDP2/GDP3. The x-axis shows the distance between the Cα of residues Phe143 and Cys249. The thin lines show the raw data and the smoother thick lines are the mobile mean of the accumulated work along 200 structures.

SUPPLEMENTARY FIGURE 1

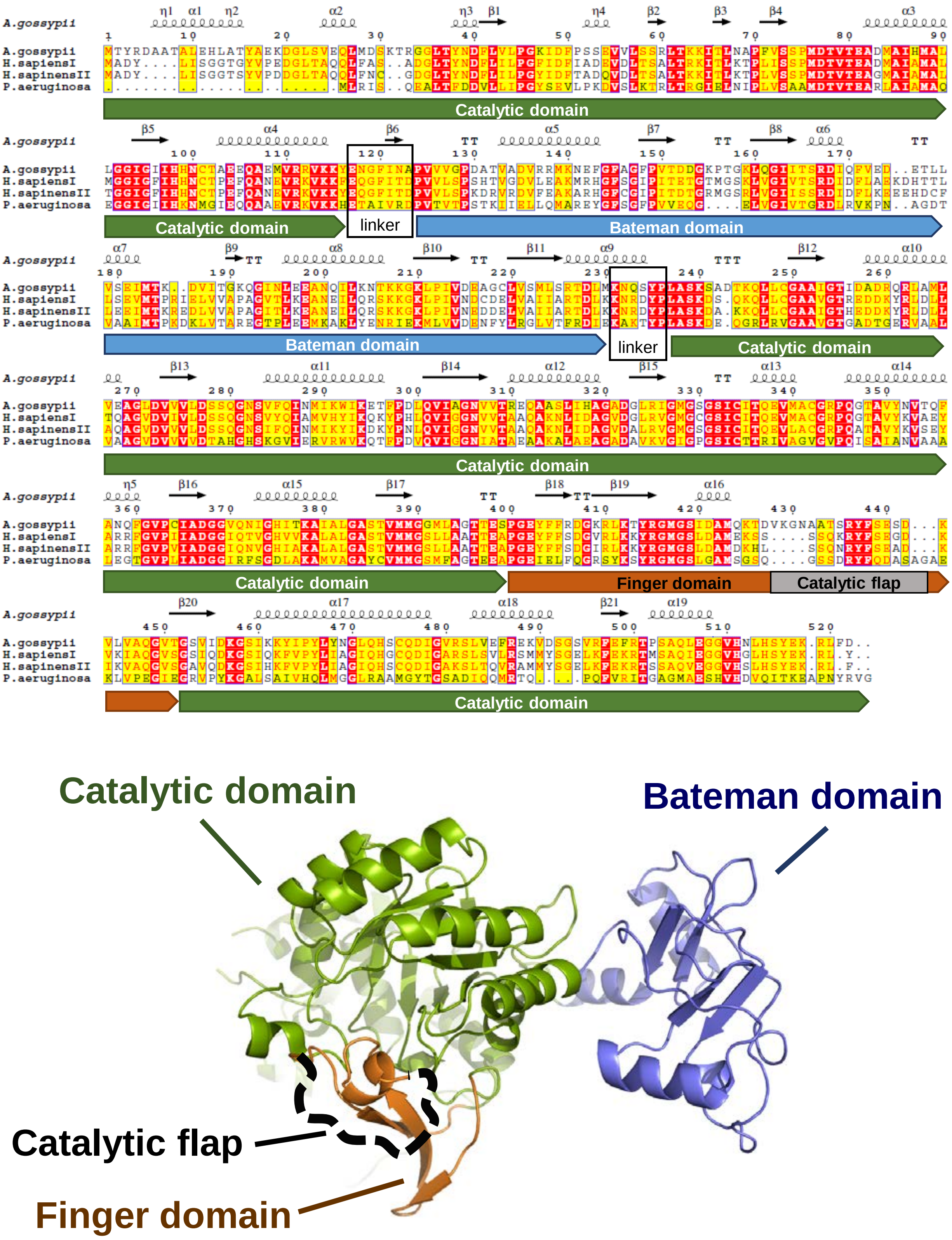

SUPPLEMENTARY FIGURE 2

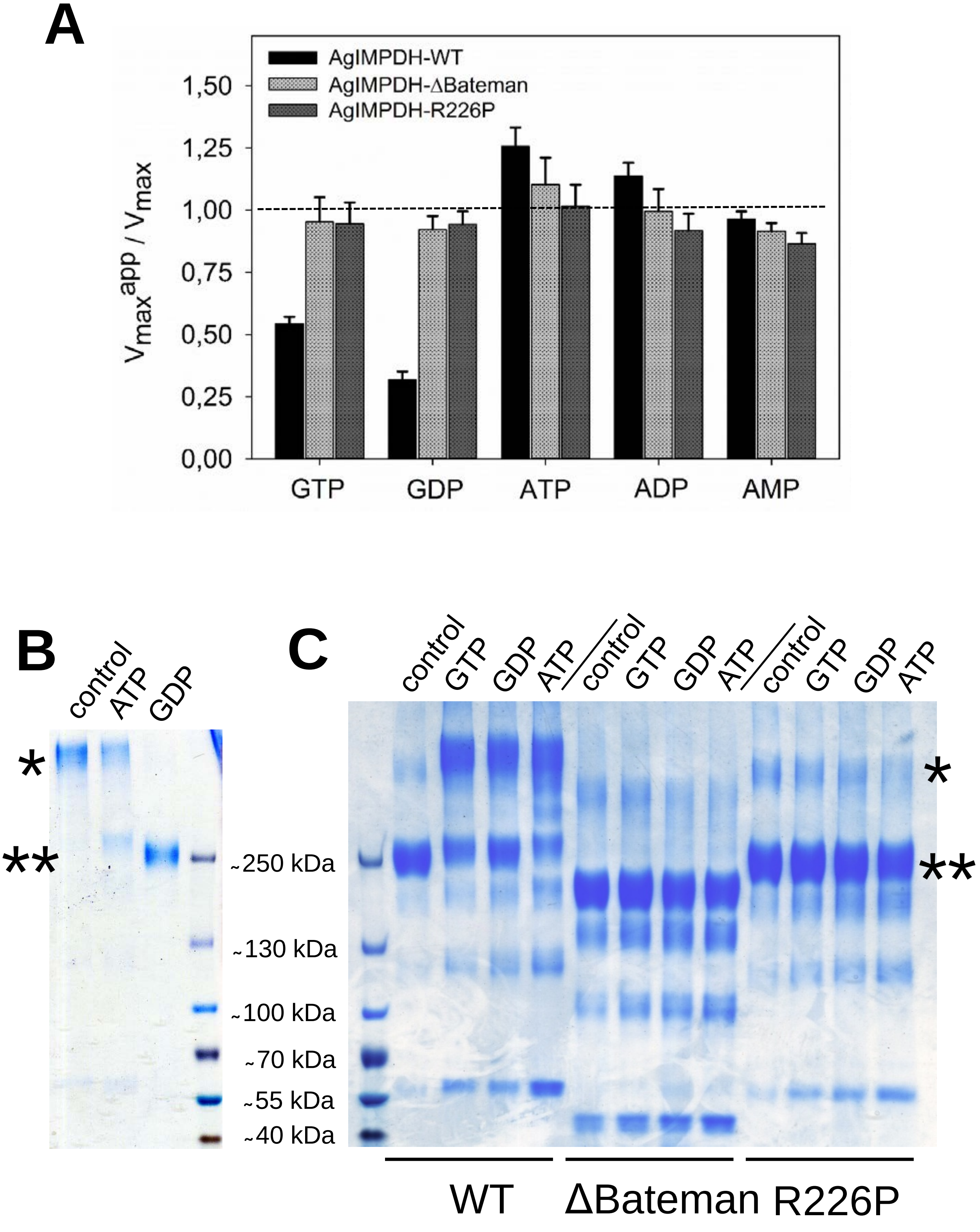

SUPPLEMENTARY FIGURE 3

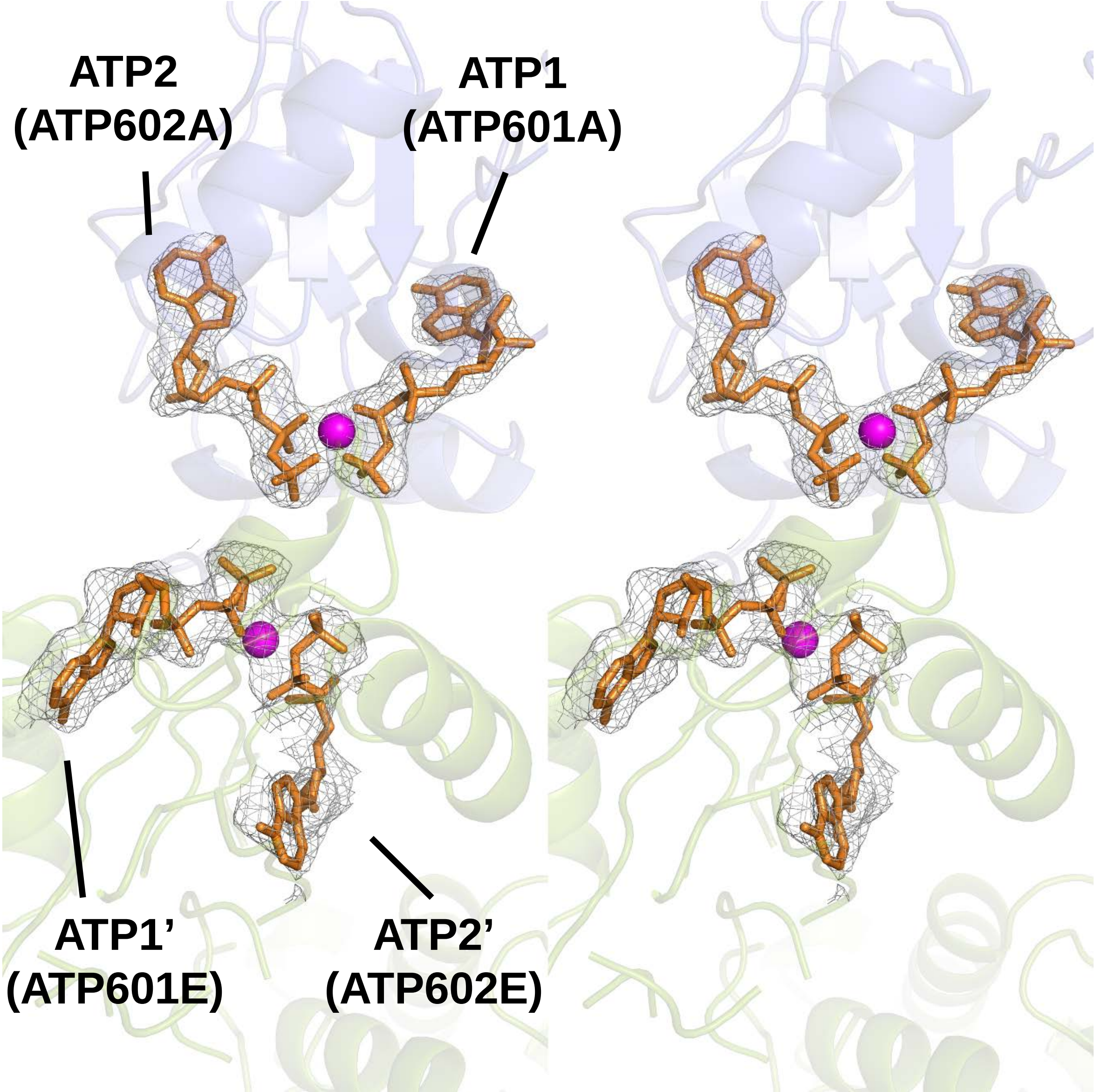

SUPPLEMENTARY FIGURE 4

A

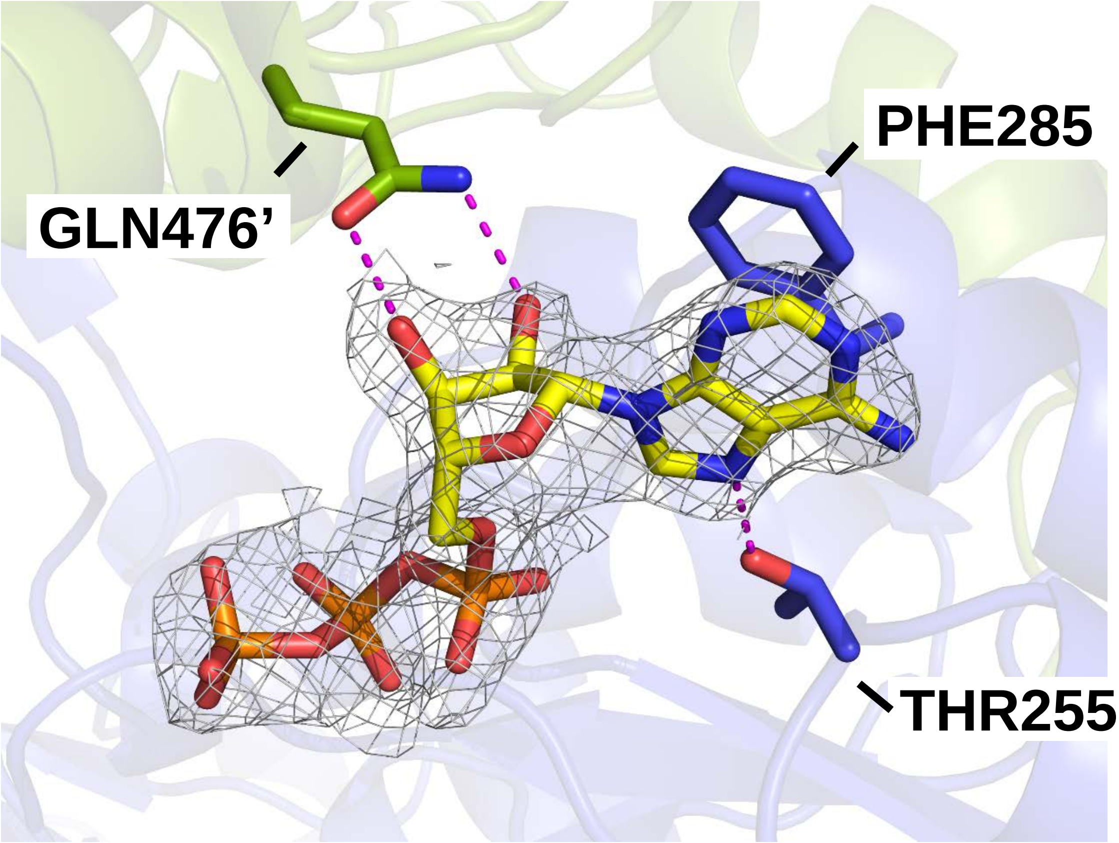

B

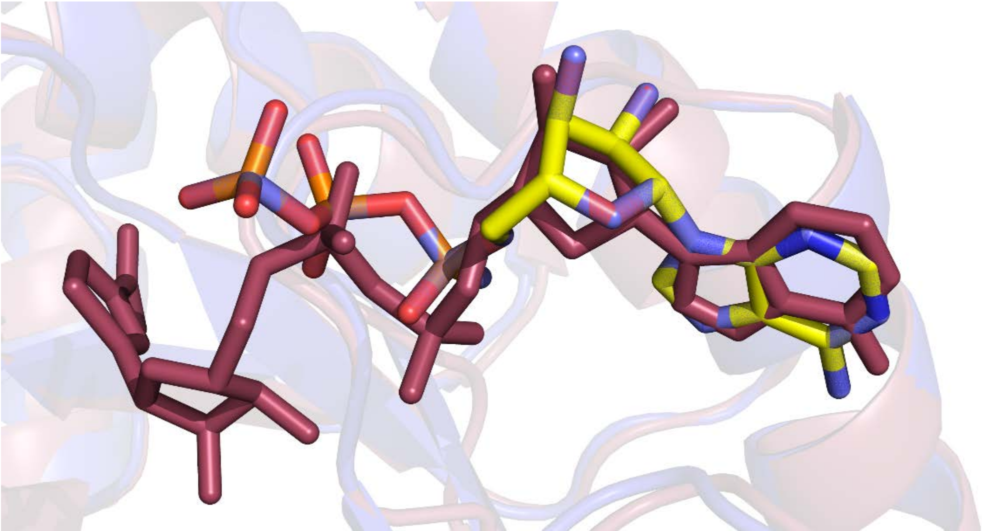

SUPPLEMENTARY FIGURE 5

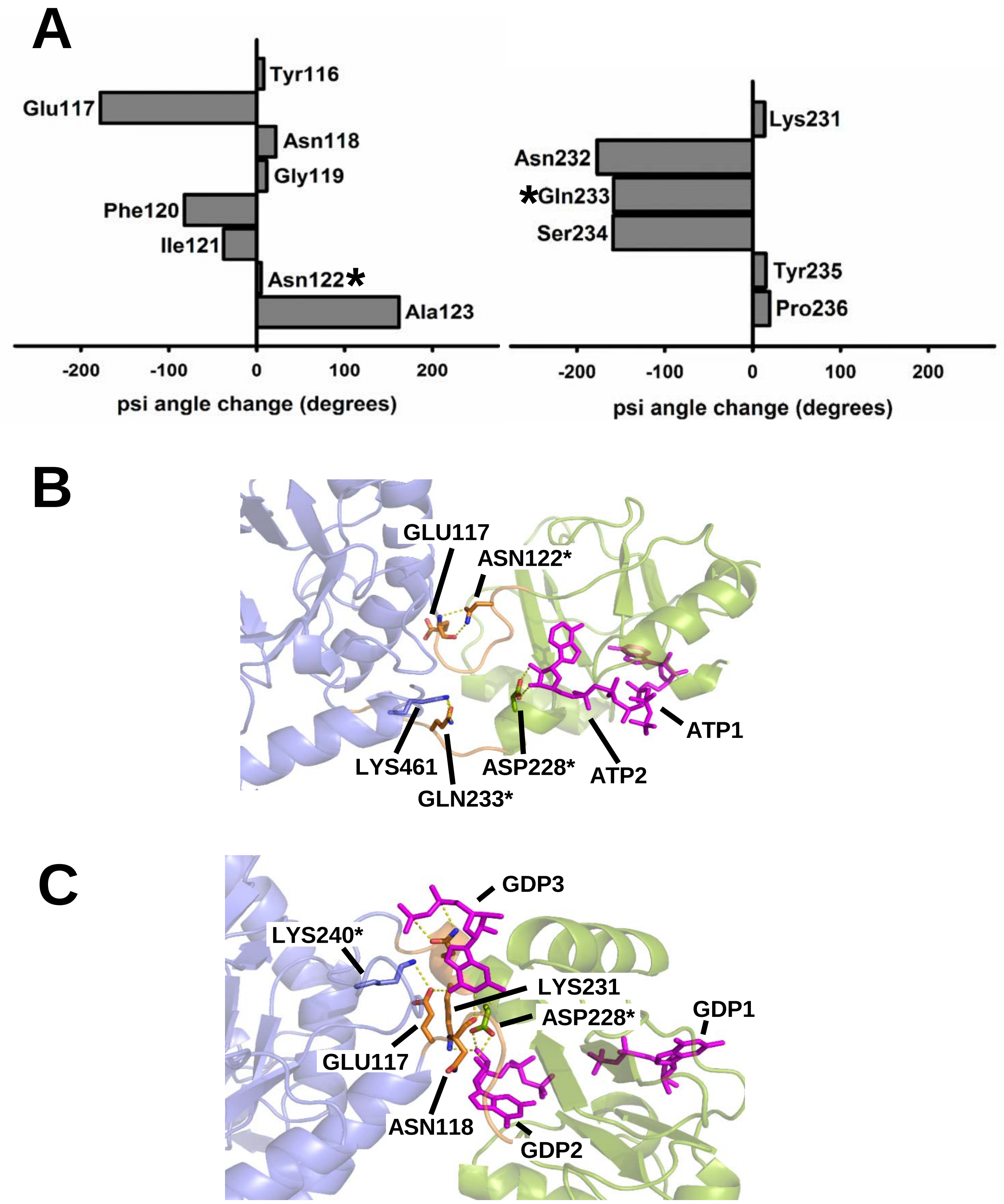

SUPPLEMENTARY FIGURE 6

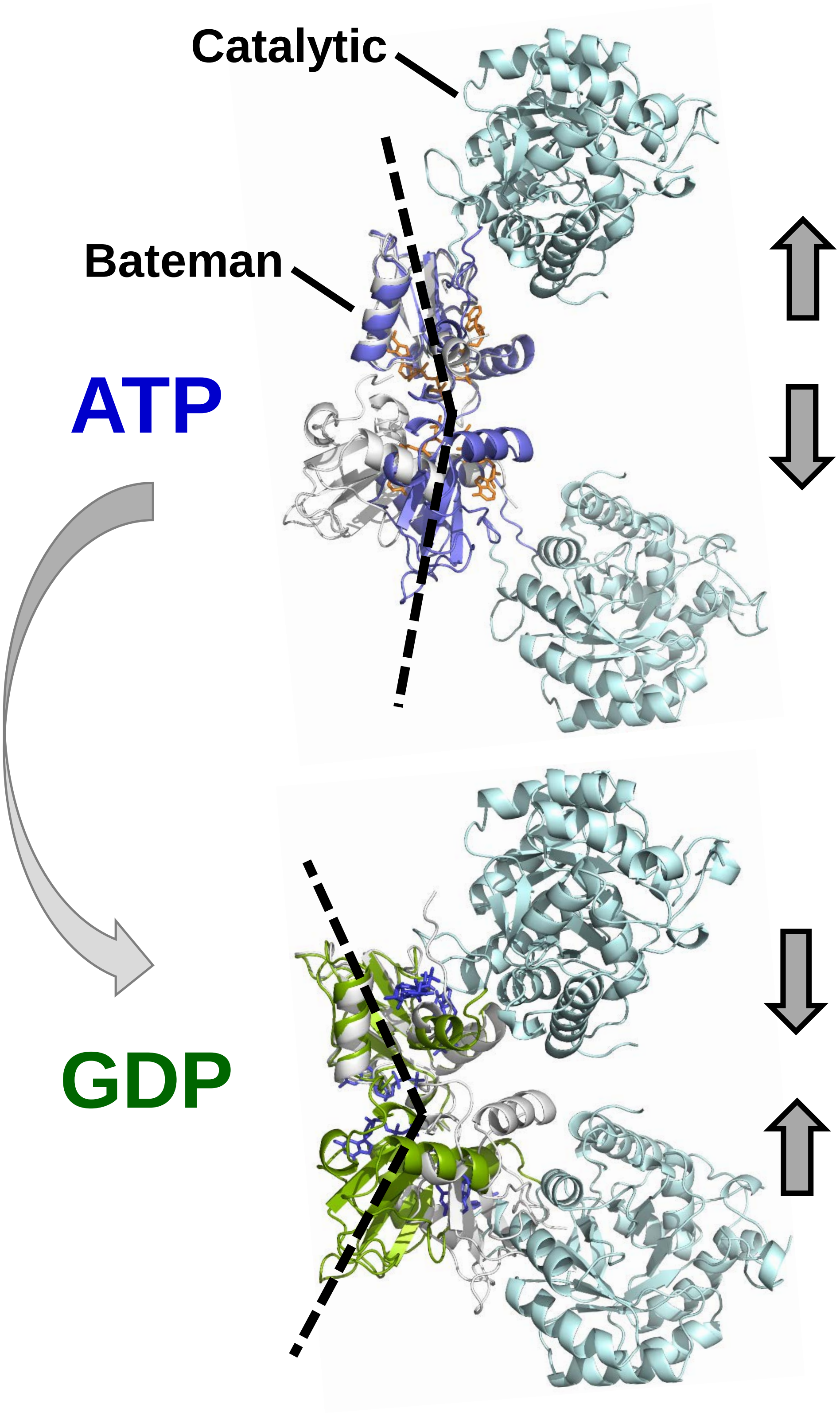

SUPPLEMENTARY FIGURE 7

A

CBS1 **CBS2**

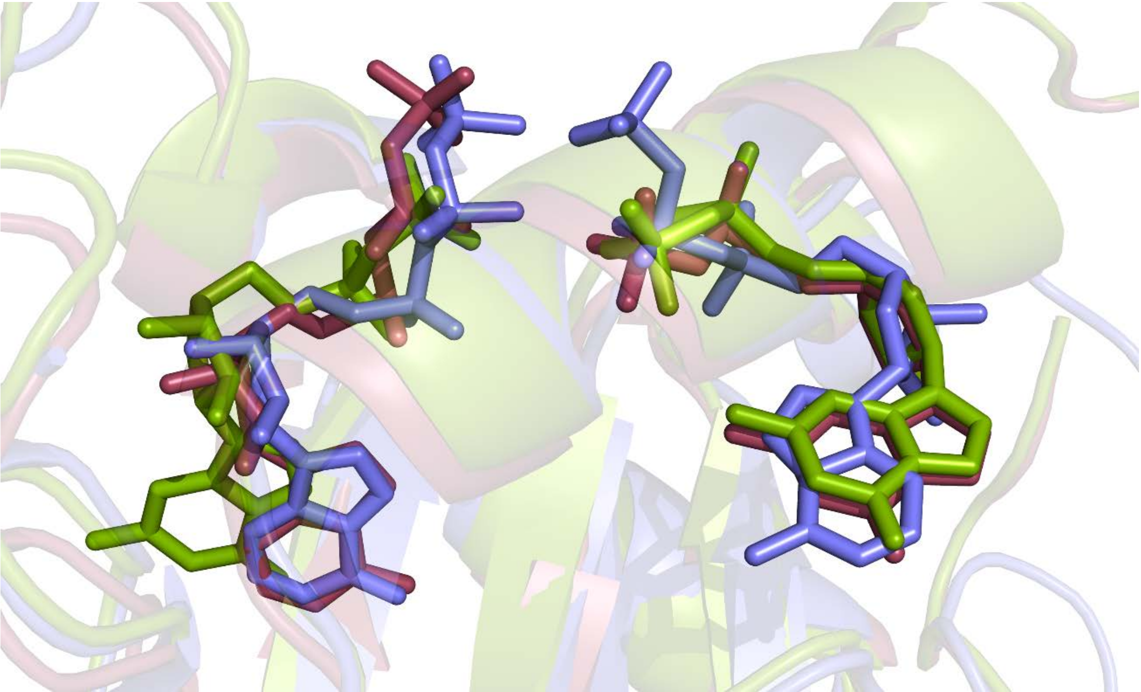

B

ATP1/ATP2 **ATP1/GDP2/GDP3** **GDP1/GDP2/GDP3**

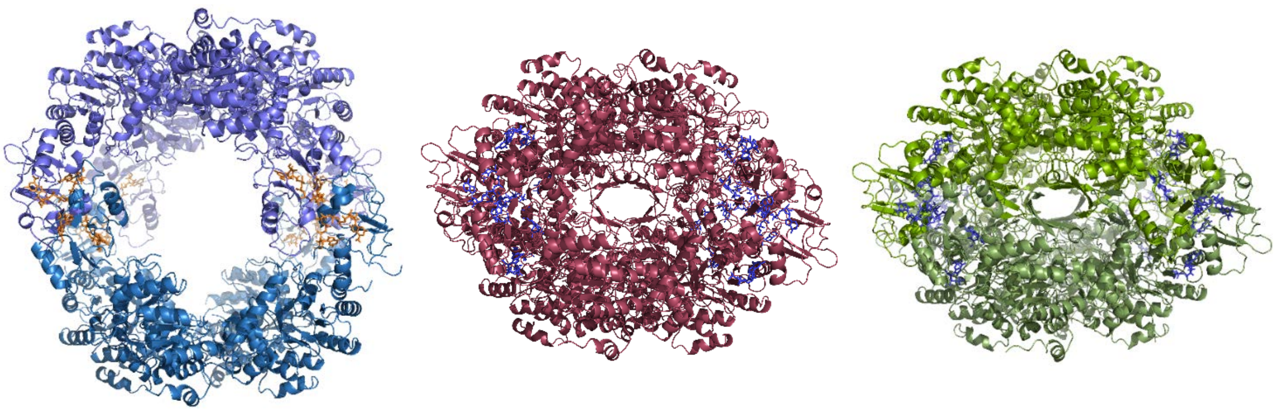

SUPPLEMENTARY FIGURE 8

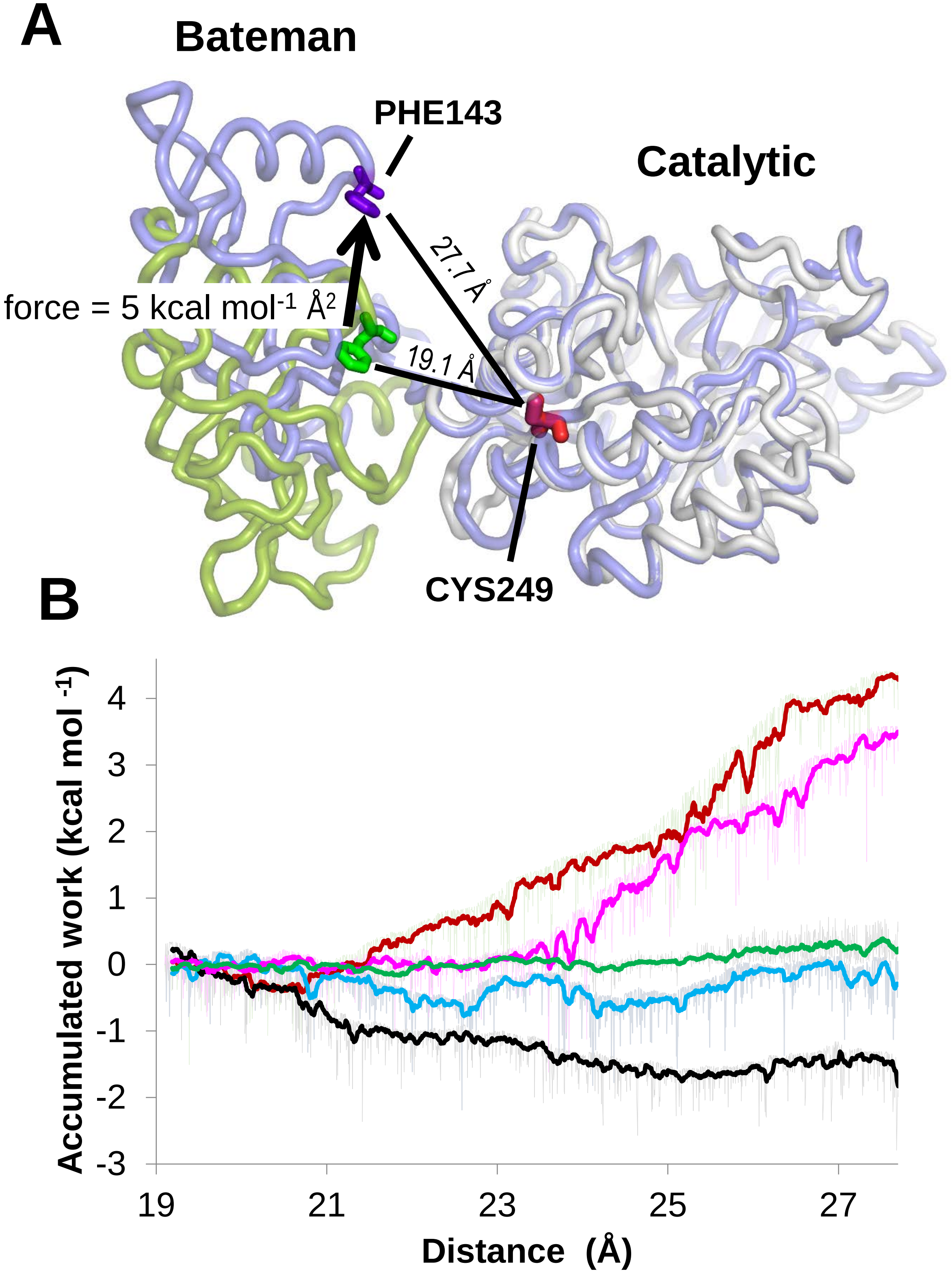

Supplement: Supplementary file 1 — Supplementary Information [file 41598_2017_2805_MOESM1_ESM.pdf]
